# DBU-intercalated γ-titanium phosphate as a latent thermal catalyst in the reaction of glycidyl phenyl ether (GPE) and hexahydro-4-methylphthalic anhydride (MHHPA)

**DOI:** 10.1039/d2ra08209h

**Published:** 2023-03-15

**Authors:** Ayumi Fujiwara, Hiroshi Furuya, Shekh Md. Mamun Kabir, Motohiro Shizuma, Atsushi Ohtaka, Osamu Shimomura

**Affiliations:** a Department of Applied Chemistry, Osaka Institute of Technology 5-16-1 Omiya, Ashahi-ku Osaka 535-8585 Japan osamu.shimomura@oit.ac.jp; b Osaka Research Institute of Industrial Science and Technology 1-6-50 Morinomiya, Joto-ku Osaka 536-8553 Japan; c Department of Wet Process Engineering, Bangladesh University of Textiles Tejgaon Dhaka-1208 Bangladesh

## Abstract

The capabilities and performance of γ-titanium phosphate (γ-TiP) with 1,8-diazabicyclo[5.4.0]undec-7-ene (DBU) as a latent thermal catalyst were investigated by the copolymerization of glycidyl phenyl ether (GPE) and hexahydro-4-methylphthalic anhydride (MHHPA) at different temperatures for a period of one hour. Polymerization was not observed until the reactants were heated to 100 °C. Upon increasing the temperature to 120 °C, the conversion in the presence of γ-TiP·DBU as a catalyst showed 98% conversion in 1 h. The thermal stability of GPE and MHHPA reacted in the presence of γ-TiP·DBU at 40 °C for 144 h resulted in less than 7% conversion of GPE. The conversion of GPE did not show a significant increase at 40 °C.

## Abstract 2

The catalytic performance of γ-TiP·DBU was investigated by copolymerization of GPE and MHHPA. The catalyst γ-TiP·DBU showed good performance as a latent thermal catalyst for storage stability and high reactivity in the reaction of GPE-MHHPA.

## Graphical abstract

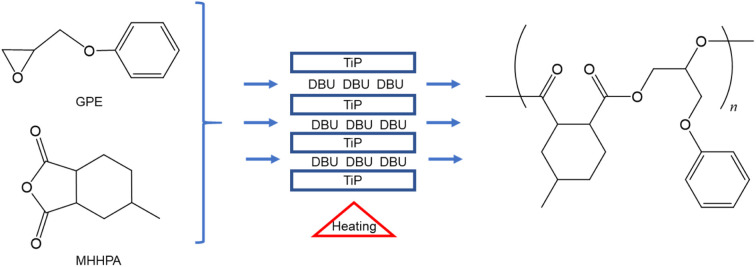

## 1. Introduction

The nanolayered structures of α-M(HPO_4_)_2_·H_2_O and γ-M(HPO_4_)_2_·2H_2_O (M = Ti, Zr, Sn, Ge, Pb, *etc.*) have attracted increasing attention in recent decades due to their ion exchange capacity and application in drug delivery.^1–5^ α-Zirconium phosphate (α-ZrP) with a small metal cation or interlaced with an amine can provide a larger interlayer distance for the further uptake of species such as a metal cation with a large ionic radius, an alkanol/glycol, and a quaternary ammonium cation, which can be considered as a catalyst and reinforcement of polymers.^6–10^ Thermal latent catalysts and initiators are highly attractive for use in chemical industries such as adhesives, paints, and molding materials. Salt-type and non-salt-type thermal latent initiators such as sulfonium salt, phosphonium salt, pyridinium salt, *N*-heterocyclic carbene, aminimide, phosphonamidates, *O*,*O*-di-*t*-butyl phenyl phosphonate and phosphonic amide ester are used in polymerization.^11^ We have already reported that primary alkylamines intercalated with α-ZrP can serve as latent thermal initiators in the reaction of glycidyl phenyl ether (GPE)^12^ and that 1,4-diazabicyclo (2,2,2) octane (DABCO) and 1,8-diazabicyclo (5,4,0) undec-7-ene (DBU) are intercalation compounds with α-ZrP.^13^ Furthermore, we examined the performances of some imidazoles intercalated with α-ZrP as thermal latent initiators.^14,15^ α-ZrP·DABCO and α-ZrP·DBU show good performance as latent thermal catalysts in the reaction of GPE and hexahydro-4-methylphthalic anhydride (MHHPA). However, α-ZrP-intercalated DBU needs a higher temperature for the reaction of GPE and MHHPA. Therefore, researchers mainly focused on developing a highly active latent catalyst to avoid the need for high-temperature curing. γ-Insoluble acid salts are tetravalent metals that can be obtained with a different layered structure, first obtained by Clearfield.^1^ Furthermore, the layers of γ-titanium phosphate (γ-TiP) and α-ZrP are packed at different interlayer distances, and hence, they show different ion exchange properties.^1^ The interlayer distance of α-Zr (HPO_4_)_2_·H_2_O (7.6 Å) and γ-Ti (HPO_4_)_2_·2H_2_O (11.6 Å) has been reported.^16^ The wide interlayer distance of γ-TiP might show high reactivity compared with α-ZrP. γ-TiP can be easily prepared by converting amorphous TiP with the treatment of concentrated H_3_PO_4_ at 225 °C for 48 h.^17^

In the present study, we report that γ-TiP intercalated with DBU was synthesized. This material could be a cheaper and alternative thermal latent catalyst than α-ZrP for the reaction of GPE and MHHPA. Therefore, the enhancement effects of γ-TiP as a potential thermal latent catalyst on the acceleration of the reaction between GPE and MHHPA were extensively studied.

## 2. Results and discussion

**Fig. 1:**
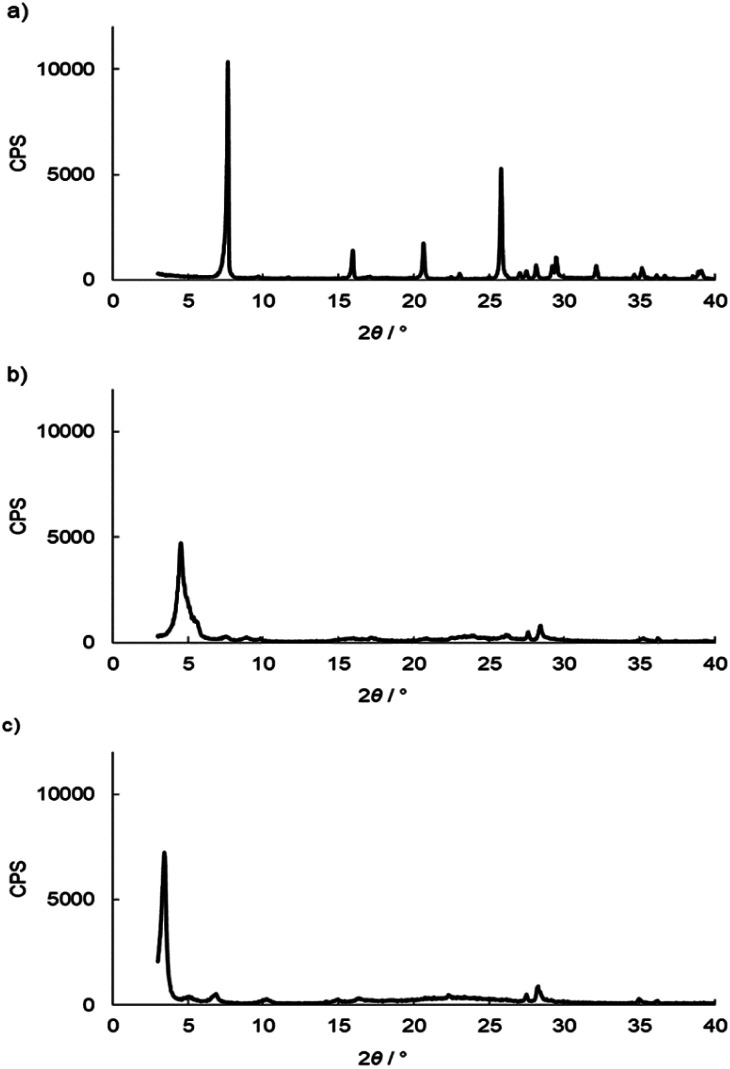
XRD patterns of (a) pristine γ-TiP, (b) γ-TiP·DBU, and (c) γ-TiP·DBU-RXN.

**Fig. 2:**
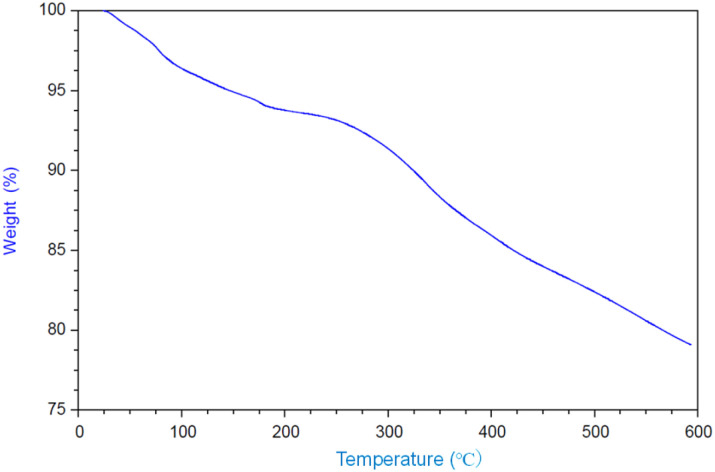
TGA curve of γ-TiP·DBU.

**Fig. 3:**
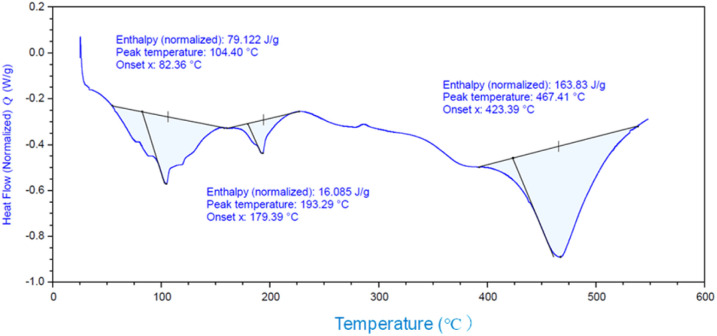
DSC curve of γ-TiP·DBU.

**Scheme 1:**
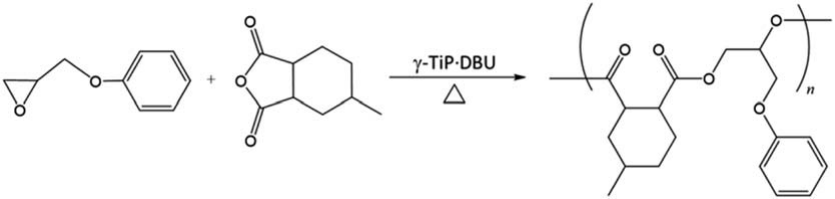
Synthesis of poly(GPE-*co*-MHHPA).

The intercalation of DBU into the layers of γ-TiP was carried out by a similar procedure of intercalation into α-ZrP. The mixture of DBU and γ-TiP in methanol was stirred at ambient temperature for 24 h. After the reaction, the intercalation compound was recovered by centrifugation and dried under vacuum. The ratio of C, H, and N of the product was 14.81%, 2.60%, and 3.84%, and the composition was Ti(HPO_4_)_2_·0.40DBU, as determined by elemental analysis. The interlayer distance of the intercalation compound of γ-TiP·DBU was 19.6 Å (2*θ* = 4.5°) expanded from 11.5 Å (2*θ* = 7.7°) of pristine γ-TiP estimated by XRD patterns, as shown in Fig. 1(a) and (b). The thermal properties of γ-TiP·DBU were examined by TG and DSC, as shown in Fig. 2 and 3. Pristine γ-TiP loses two molecules of crystal water from 50 to 100 °C.^16^ As shown in Fig. 2, γ-TiP·DBU gradually lost the weight in the first step until 220 °C (6.5% of weight loss). The DSC curve up to 220 °C shows two endothermic peaks, and the peak temperatures are 104.4 °C and 193.3 °C, as shown in Fig. 2. The third endothermic peak was observed at 423.4 °C from 300 °C to 550 °C. Assuming that the weight loss of 20.8% from 25 °C to 590 °C was attributed to that of DBU, the compositional formula was calculated as Ti(HPO_4_)_2_·(C_9_H_16_N_2_)_0.41_. From the compositional formula calculated from the elemental analysis, the composition was Ti(HPO_4_)_2_·(C_9_H_16_N_2_)_0.40_. The decreasing weight was in good accordance with the deintercalation of DBU. It could be explained that γ-TiP shows a two-step titration curve with a NaOH–NaCl solution.^1^ At least two peaks would be indicated in the DSC curve. The reaction of GPE–MHHPA with γ-TiP·DBU, as shown in Scheme 1, was carried out at 120 °C for 4 h, the polymer products obtained were washed out with THF and the residue of γ-TiP·DBU (γ-TiP·DBU·RXN) was recovered. The interlayer distance of γ-TiP·DBU·RXN was 25.7 Å (2*θ* = 3.4°) expanded from γ-TiP·DBU, as shown in Fig. 1(c).

**Fig. 4:**
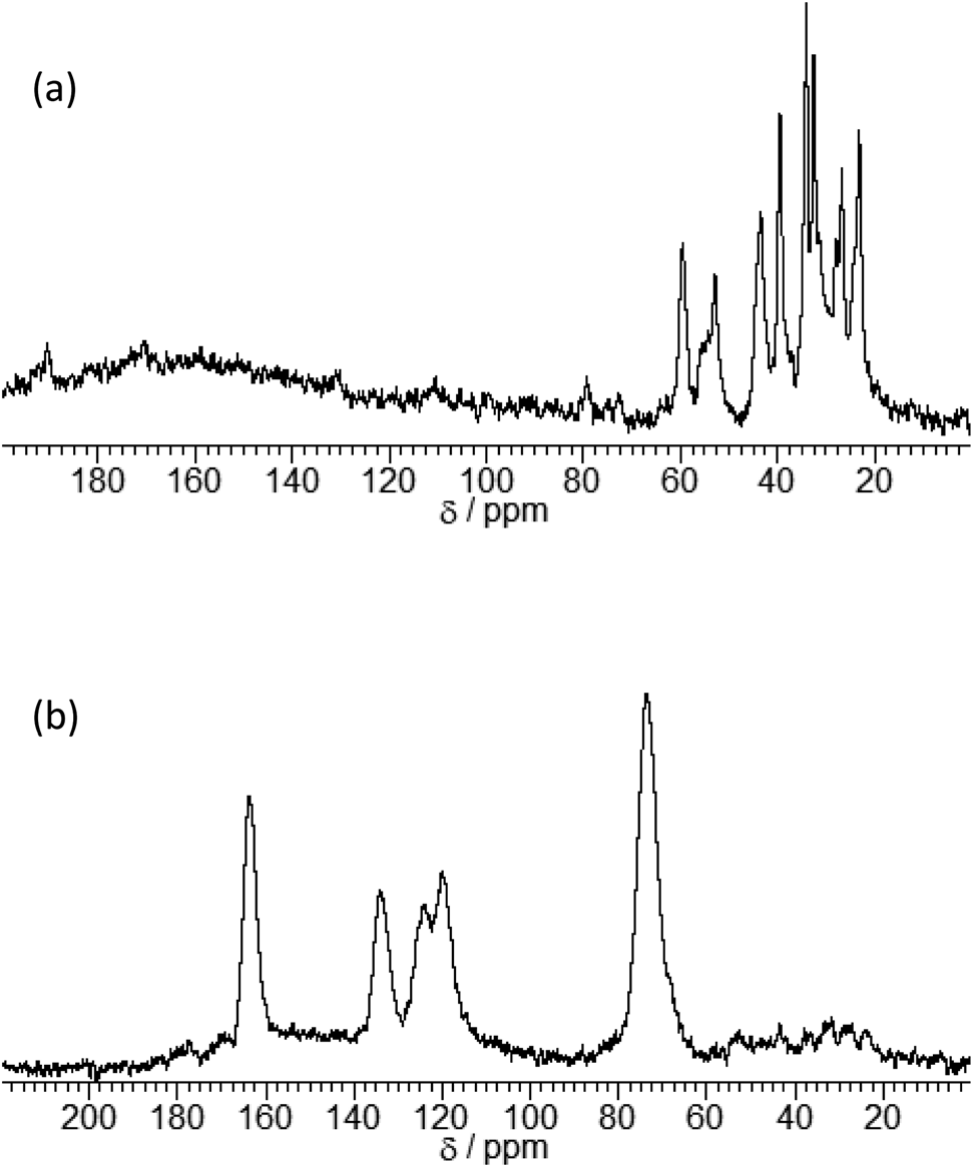
^13^C-NMR spectra of (a) γ-TiP·DBU and (b) γ-TiP·DBU-RXN.

**Fig. 5:**
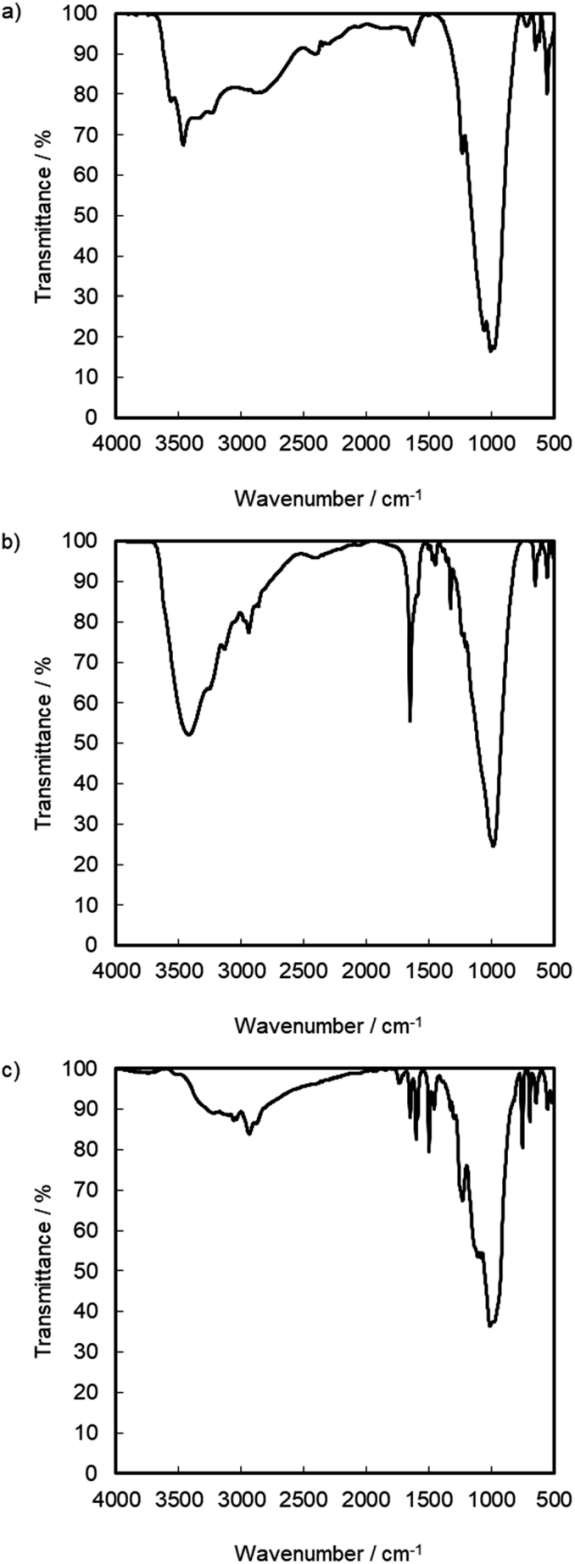
FT-IR spectrum of (a) pristine γ-TiP, (b) γ-TiP·DBU, and (c) γ-TiP·DBU-RXN.

The expansion of the basal distance and an increase in the C content were recognized. The ^13^C CPMAS NMR spectrum of γ-TiP·DBU and γ-TiP·DBU·RXN are shown in Fig. 4. In the ^13^C CPMAS NMR spectrum, the aromatic carbons, methine, and methylene groups derived from GPE are shown in Fig. 4(b). The copolymer of GPE-MHHPA, carbonyl carbon, must be observed at *δ* 175. In the ^13^C CPMAS NMR of γ-TiP·DBU-RXN, carbonyl carbon was not observed at *δ* 175. A similar result was observed with α-ZrP imidazoles.^14^ The C, H, and N ratio of the product by elemental analysis was 33.03 : 3.46 : 1.28. Based on the result, the reaction products exist in the interlayer of γ-TiP·DBU, and the composition was estimated as Ti(HPO_4_)_2_·DBU_0.20_·(GPE)_1.15_. At 50%, the interlayer of DBU remained after the reaction of GPE-MHHPA. We have already reported using α-ZrP·DBU that the remaining DBU was 20%.^13^ The deintercalated DBU initiated the copolymerization of GPE and MHHPA. The deintercalation ratio would be affected by the reactivity of GPE-MHHPA. The FT-IR spectra of α-ZrP·DBU and α-ZrP·DBU-RXN are shown in Fig. 5(b) and (c). The peak due to DBU (*ν* C=N) and α-ZrP (*ν* P–O) was detected at 1650 and 983 cm^−1^ in Fig. 5(b). The aromatics (*ν* C–C at 1600 and 1548 cm^−1^) and ether groups (*ν* C–O–C at 1230 cm^−1^) in the products of GPE are observed in Fig. 5(c).

**Fig. 6:**
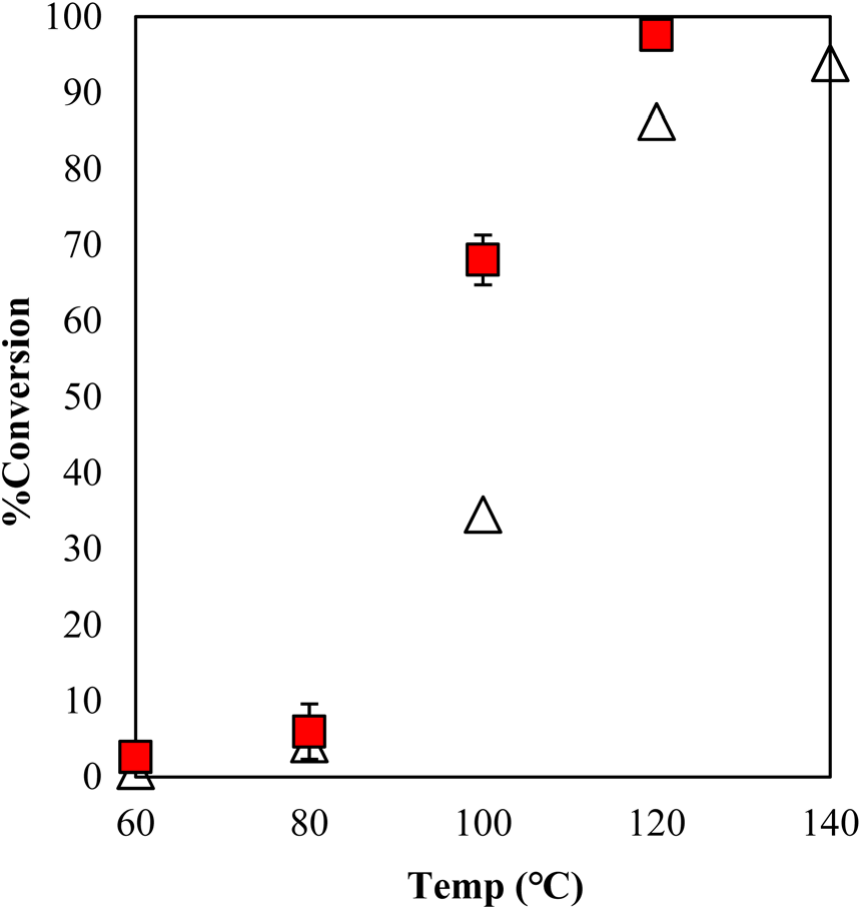
Conversion of GPE after 1 h as a function of temperature during polymerization with γ-TiP·DBU (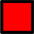) and α-ZrP·DBU (Δ).^13^

The estimation of the catalytic activity of γ-TiP·DBU and the copolymerization of GPE and MHHPA was carried out as shown in Fig. 6. The reaction of GPE-MHHPA with α-ZrP·DBU has already been reported.^13^ In comparison to GPE conversions at 100 °C, the greatest difference between the conversions was 68% and 35% with γ-TiP·DBU and α-ZrP·DBU, as determined by the ^1^H-NMR spectra. Conversions at 120 °C were 98% and 86% with γ-TiP·DBU and α-ZrP·DBU. The reaction with γ-TiP·DBU quantitatively proceeded at 120 °C and γ-TiP·DBU was higher reactivity at the same reaction temperature.

**Fig. 7:**
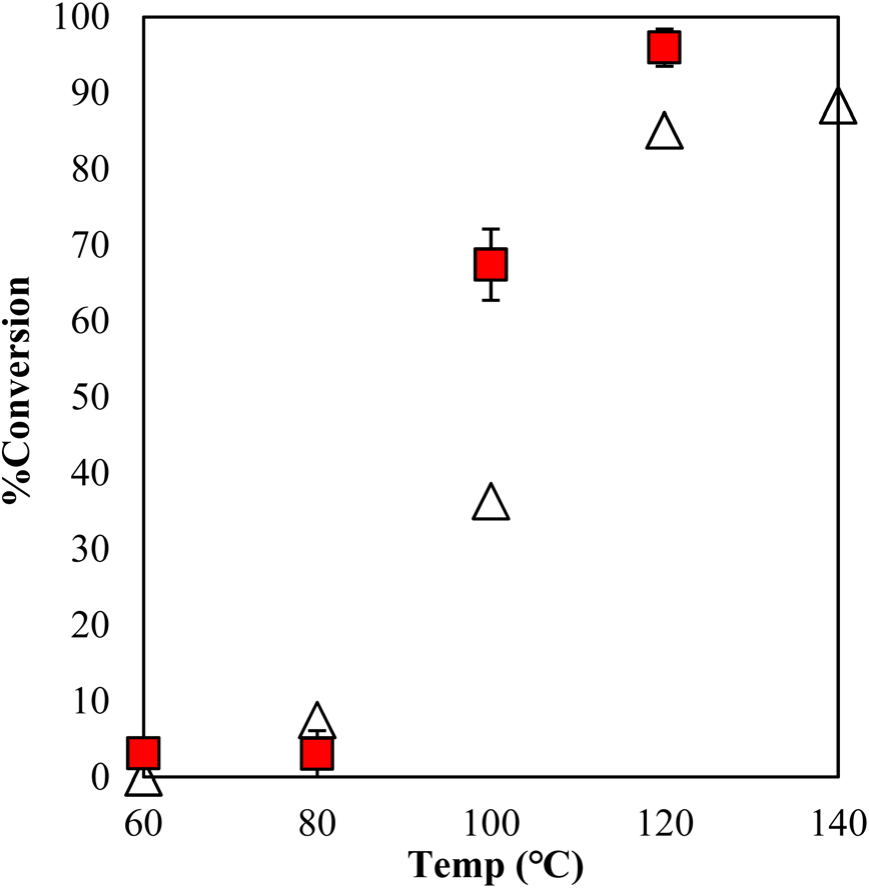
Conversion of MHHPA after 1 h as a function of temperature during polymerization with γ-TiP·DBU (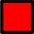) and α-ZrP·DBU (Δ).^13^

The copolymerization of GPE-MHHPA and homopolymerization of GPE might occur simultaneously. The conversion of MHHPA was confirmed by the integral ratio at different temperatures for 1 h (Fig. 7). However, γ-TiP·DBU has shown a conversion of 67% at 100 °C and increased the conversion rate by 96% at 120 °C. It can be explained that the conversion of GPEs was in good accordance with MHHPA and γ-TiP·DBU accelerates the reaction rate more than α-ZrP·DBU. The reaction with γ-TiP·DBU quantitatively proceeded at 120 °C and γ-TiP·DBU has higher reactivity at the same reaction temperature. It might be explained that γ-TiP shows a two-step titration curve with the NaOH–NaCl solution. Under a similar condition, α-ZrP shows a one-step titration curve, as reported in the literature.^1^ It can affect the first step of deintercalation of DBU from the interlayer of γ-TiP.

**Fig. 8:**
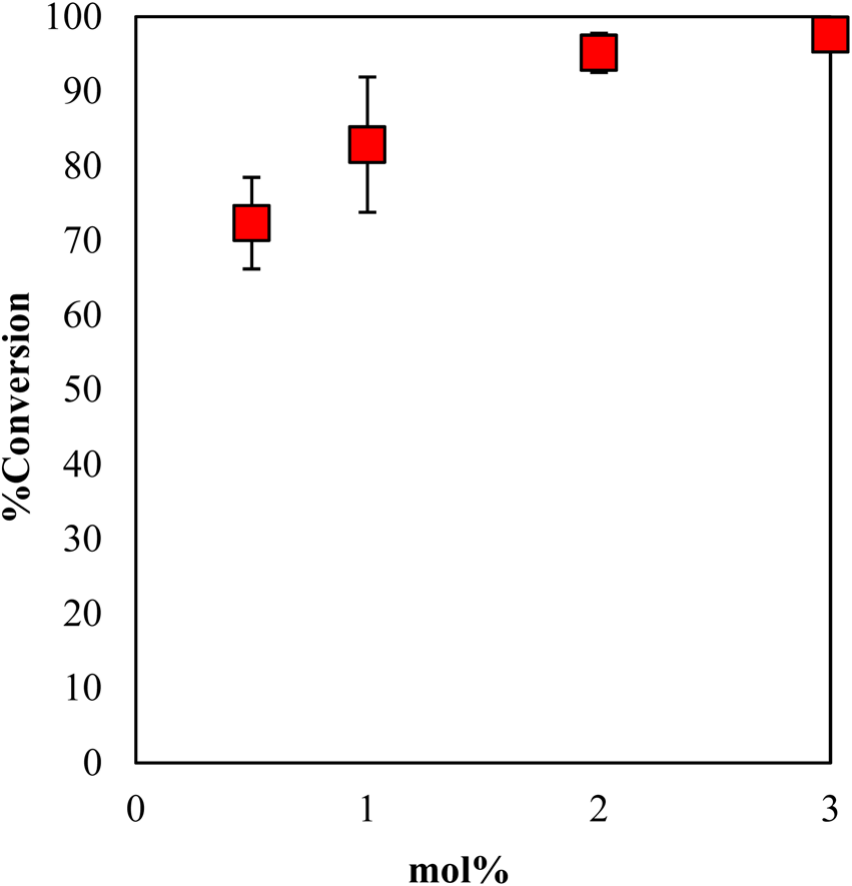
Conversion of GPE after 1 h as a function of the content of DBU in γ-TiP·DBU during polymerization at 120 °C.

The conversion of GPE after 1 h as a function of the content of DBU in γ-TiP·DBU during polymerization is shown in Fig. 8. When the mole concentration of DBU increased, and the conversion rate also increased. The comparison of the conversions of GPE was 95% by using 2 mol% of γ-TiP·DBU. There is also a very important observation in the presence of γ-TiP·DBU; the use of more than 2 mol% γ-TiP·DBU resulted in copolymerization of GPE and MHHPA.

**Fig. 9:**
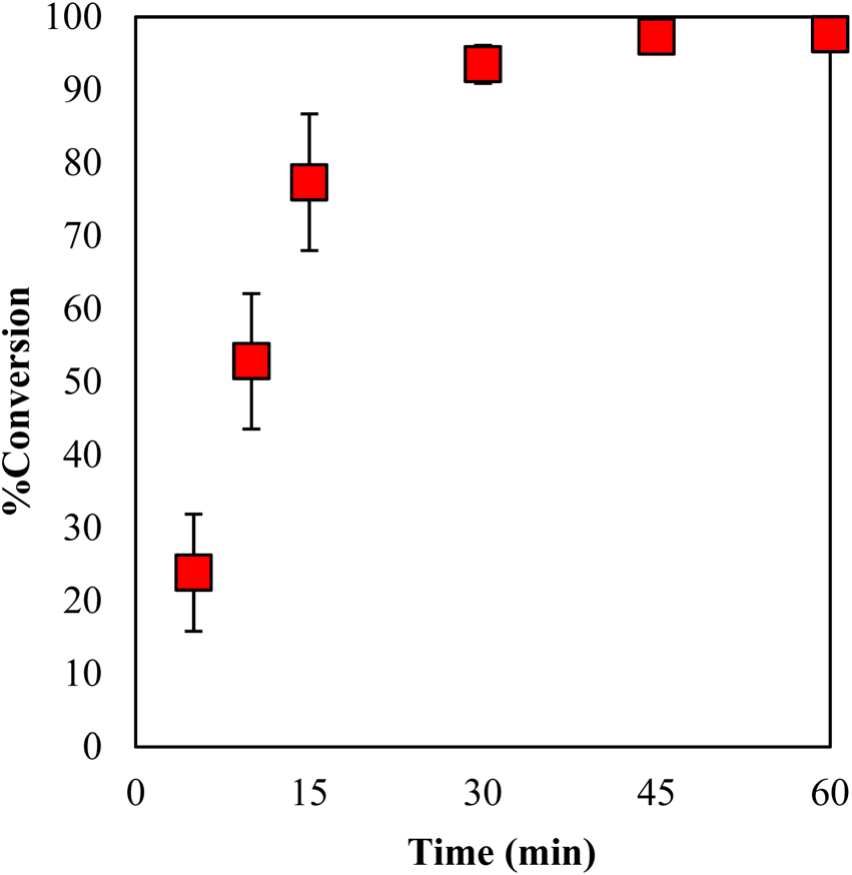
Conversion of GPE as a function of time during polymerization with γ-TiP·DBU at 120 °C.

The conversion values for GPE with 3 mol% of γ-TiP·DBU at 120 °C for 0–60 min are displayed in Fig. 9. It can be seen that the conversion values increased with the increasing reaction time and reached 94% after 30 min. It is clear that the polymerization reaction proceeds within a short period after 30 min under specific conditions. In addition, by extending the time period, the conversion rate also increased and reached 97% conversion for 45 min.

**Fig. 10:**
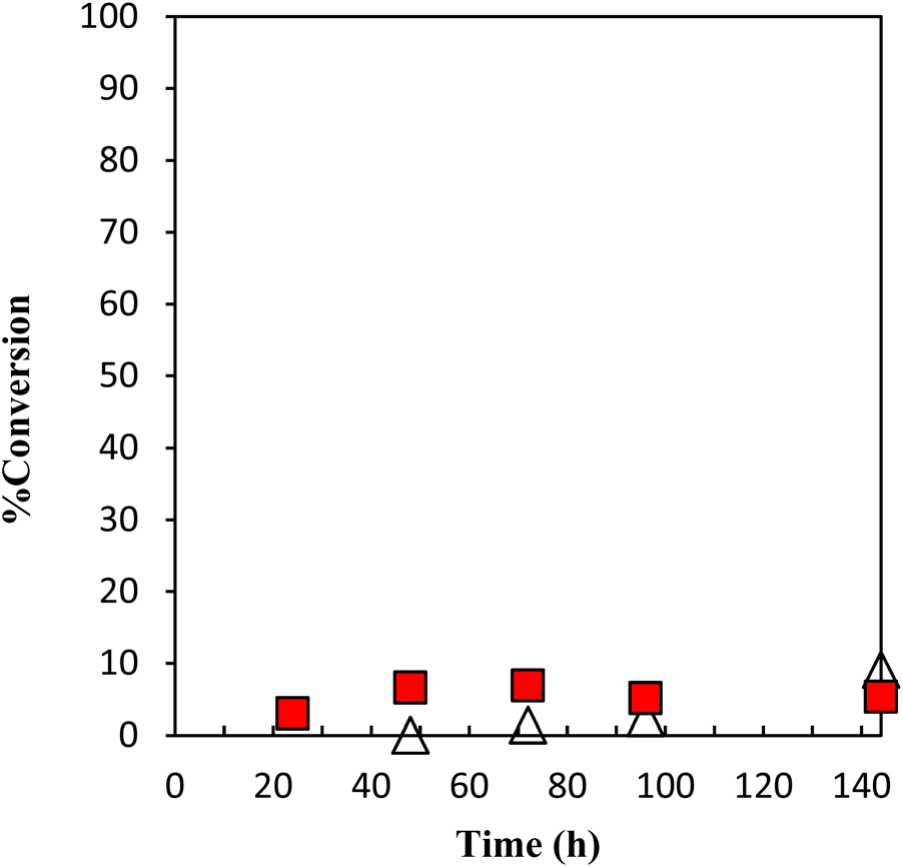
Conversion of GPE as a function of time during polymerization with γ-TiP·DBU at 40 °C. γ-TiP·DBU (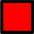) and α-ZrP·DBU (Δ).^13^

The performance of the latent thermal initiator depends on the high curing capacity at the desired temperature and higher storage stability under the recommended storage conditions. The evaluation of the performance of storage stability is mainly based on the conversion of GPE as a function of time during polymerization with γ-TiP·DBU and α-ZrP·DBU at 40 °C, as presented in Fig. 10. The conversion did not show a significant increase, and the conversion was 5% for 144 h.

## 3. Experimental section

### 3.1. Materials

Ti(HPO_4_)_2_·2H_2_O was purchased from Rasa Industries Ltd (Japan); GPE, DBU, and MHHPA from Tokyo Chemical Industries, Co., Ltd (Japan). Solvents were used as received without further purification.

### 3.2. Measurements

X-ray diffraction (XRD) patterns were acquired using a Rigaku RINT2200 (Japan) with Cu Kα radiation over a scan range of 3–40° at a rate of 2° min^−1^. NMR spectra in solutions were recorded using a Varian Unity-300 spectrometer (Palo Alto, CA, USA) with tetramethylsilane (TMS) as an internal standard. The DBU contents in the intercalation compounds of γ-TiP were measured using a PerkinElmer 2400II analyzer (Waltham, MA, USA). The ^13^C CPMAS NMR spectra were recorded using a JEOL ECA-600 NMR spectrometer (Tokyo, Japan). Thermogravimetric (TG) analysis was carried out using a TA instrument TGA-550 at a heating rate of 10 °C min^−1^ under nitrogen. Differential scanning calorimetry (DSC) was carried out using a TA instrument DSC250 at a heating rate of 10 °C min^−1^ under nitrogen.

### 3.3. Preparation of DBU-intercalated γ-TiP (γ-TiP·DBU)

The intercalation of DBU into the layers of Ti(HPO_4_)_2_·2H_2_O (γ-TiP) was carried out following a similar procedure for α-Zirconium phosphate. γ-TiP (4.98 g) and DBU (8.38 g) were added to methanol (75.2 mL). The reaction mixture was agitated at ambient temperature for 24 h before the product was collected by centrifugation and washed 3 times with methanol. The resulting residue was dried under vacuum. The ratio of C, H, and N in the product was 14.81 : 2.60 : 3.84, and the composition was TiP(HPO_4_)_2_·0.40DBU, as determined by elemental analysis.

### 3.4. Typical polymerization procedure

A mixture of GPE (150 mg, 1.0 mmol), MHHPA (166 mg, 0.99 mmol), and γ-TiP·DBU (22.1 mg, 0.075 mmol, DBU content: 0.030 mmol) was heated at 100 °C for 1 h. A small aliquot of the reaction mixture was dissolved in CDCl_3_, and its ^1^H-NMR spectrum was recorded to determine the extent of conversion of GPE.

### 3.5. Recovery of γ-TiP·DBU after the reaction with GPE and MHHPA (γ-TiP·DBU-RXN)

A mixture of GPE (7.51 g, 50.0 mmol), MHHPA (8.41 g, 50.0 mmol), and γ-TiP·DBU (535 mg, 1.82 mmol, DBU content: 0.73 mmol) was heated at 120 °C for 4 h. After the reaction, tetrahydrofuran (THF) was added to the mixture. The solution was filtered and the residue, γ-TiP·DBU-RXN, was rinsed, dried under vacuum, and analyzed by XRD and ^13^C CPMAS NMR spectra. The C, H, and N ratio in γ-TiP·DBU-RXN was 33.03 : 3.46 : 1.28.

## 4. Conclusions

A temperature-dependent latent thermal catalyst γ-titanium phosphate (γ-TiP) intercalated with DBU has been introduced in the reaction of glycidyl phenyl ether (GPE) and hexahydro-4-methylphthalic anhydride (MHHPA) and compared with α-ZrP-intercalated DBU. The interlayer distance of the intercalation compound of γ-TiP·DBU has shown 19.6 Å expanded from 11.5 Å of pristine γ-TiP. In the reaction with GPE and MHHPA at 120 °C for 1 h, the conversion value of GPE has been reached at 99% by γ-TiP·DBU. In addition, the conversion of MHHPAs was in good accordance with the conversion of GPEs. The conversion values of GPE increased with the increasing reaction time and reached 96% after 30 min and 98% after 45 min, by the reaction as a function of time during polymerization with γ-TiP·DBU. The latent thermal catalyst γ-TiP·DBU showed good stability under typical storage conditions (144 h at 40 °C), and was highly reactive with GPE and MHHPA. Thus, it can be suggested that γ-TiP·DBU could be a good alternative as a latent thermal initiating system in curing epoxy resins.

## Author contributions

Osamu Shimomura conceived, designed, and wrote the article; Ayumi Fujiwara, Hiroshi Furuya, and Motohiro Shizuma performed the experiments; Shekh Md. Mamun Kabir and Atsushi Ohtaka contributed to a helpful discussion.

## Conflicts of interest

The authors declare no conflict of interest.

## Supplementary Material
